# A case report on role of hypertonic saline solution in management of parotid fistula

**DOI:** 10.1016/j.amsu.2022.104208

**Published:** 2022-07-15

**Authors:** Himal Bikram Bhattarai, Rukesh Yadav, Sangam Shah, Manish Uprety, Ayusha Subedi, Prabesh Bikram Singh, Anirudra Devkota, Niranjan Panthi

**Affiliations:** a Gandaki Medical College, Pokhara, Nepal; b Tribhuvan University, Institute of Medicine, Maharajgunj, 44600, Nepal; c Kathmandu University of Medical Sciences, Kathmandu, Nepal; d Manmohan Memorial and Community Hospital, Jhapa, Nepal; e B.P. Koirala Institute of Health Sciences, Dharan, Nepal

**Keywords:** Parotid, Fistula, Hypertonic saline

## Abstract

**Introduction:**

Parotid fistula is an uncommon consequence of surgical or non-surgical trauma to the parotid gland or the area surrounding it. To treat it, a variety of pharmacological medicines and surgical techniques are used, each with their own set of benefits, drawbacks, and patient preferences.

**Case Presentation:**

We present the successful care of a young female with post-traumatic parotid fistula using hypertonic saline injections into the parotid substance, which is a simple yet efficient approach of treating this abnormality.

**Discussion:**

Thermodynamic and physicochemical calculations suggest that hypertonic saline solutions work to close parotid fistula by causing conformational denaturation of the cell membrane proteins in situ and saline can be diluted to a point where there will be no cellular toxicity. It is advised that temperature of the saline should be raised above body temperature to enhance the fibrosing property of physiologic saline.

**Conclusion:**

The use of hypertonic hot saline injections combined with compression dressing is a cost-effective, patient-friendly, and almost complication-free approach of treating parotid fistulas with promising results.

## Highlights

• Parotid fistula is an uncommon consequence of surgical or non-surgical trauma to the parotid gland.
• The use of hypertonic hot saline injections is a cost-effective treatment.

## 1 Introduction

Parotid fistula is a very unusual and unpleasant complication that can occur after an injury to the maxillary area of the face. Any surgery in the vicinity of the parotid gland, such as parotidectomy, or surgery surrounding the mandible and the temporomandibular joint, such as mandibular osteotomy, could be the source of harm [1,2]. Parotid fistula can develop as a result of difficulties caused by the draining of a face or parotid abscess. Parotid fistula is a well-known complication of face trauma treatment, such as the use of external pin fixation, or as a result of facial fractures [[3], [4], [5]]. Several conservative and aggressive treatment techniques with varying degrees of success and morbidity have been described. In this work, we describe how a young female with a post-traumatic parotid fistula was successfully treated with hypertonic saline injections into the parotid material, a simple yet effective method of treating this abnormality. This case was reported in accordance with SCARE 2020 requirements [6].

## 2 Case Presentation

**Fig. 1:**
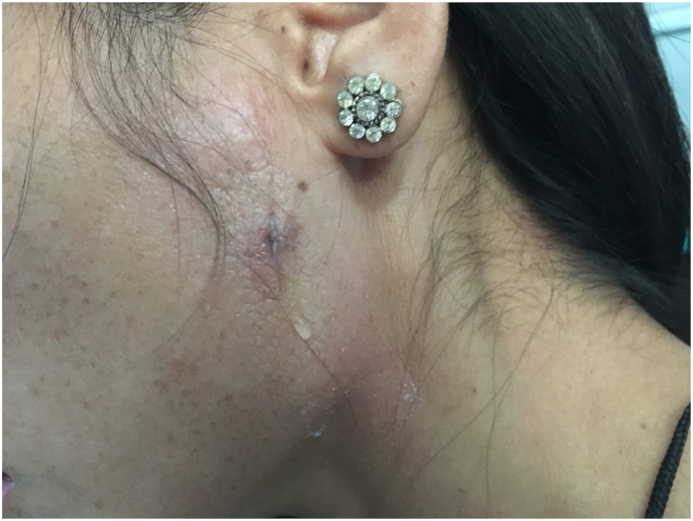
Watery discharge from the parotid fistula.

A female of age 24 presented to the emergency department with a traumatic cut injury over her cheek. The cut injury was sutured in emergency department, and she was discharged with appropriate medication. She came back for a follow-up visit for suture removal. Four days after suture removal, the patient presented with a watery, odorless discharge coming from the previously sutured surgical wound (Fig. 1).

A diagnosis of the parotid fistula was made based on history, location, and clinical inspection of the discharge. We tried to manage the patient with pressure dressings, anti-sialagogues and antibiotics. There was no significant decrease in the size of swelling or discharge. The patient still complained of watery discharge from the fistula.

Afterward, we decided to use hot hypertonic saline injections. Five milliliters of 3% hypertonic solution prepared in autoclave at temperature of 60° were injected into the parotid through the fistulous opening. The patient was instructed to continue the same medicines. This procedure was continued for four consecutive days. On the fifth day, the patient did not show any signs of swelling and salivary leak suggesting closure of parotid fistula.

Upon follow-up visit till few months the patient was fine with no complaints. The facial nerve and its branches were evaluated and were found to be normal. The function of facial nerve was intact as well.

## 3 Discussion

**Fig. 2:**
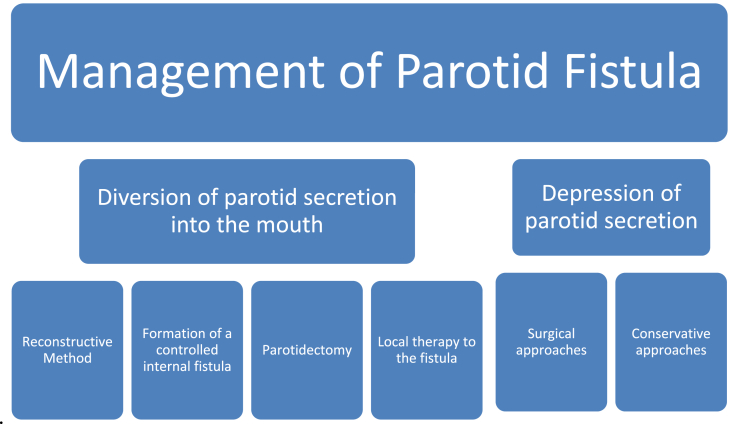
Management of Parotid fistula.

The treatment of parotid fistula has been controversial and disappointing in the past. A number of procedures are described, each with a different success rate and morbidity. As demonstrated in Fig. 2, surgical or conservative methods are commonly used to treat it. Surgical methods that channel parotid secretions towards the mouth and those that restrict parotid secretion through ductal ligation or nerve sectioning can be split into two categories. As part of a conservative strategy, anti-sialagogues or radiation may be employed to try to suppress secretion [7,8]. This study goes over the many treatment options for parotid fistulas, with hypertonic saline being the most commonly used. We used heated hypertonic saline injections into the glandular material in our patient with parotid fistulae to induce rapid gland fibrosis.

The primary challenge with reconstructive surgical techniques that aim to divert secretions into the mouth is the extensive scarring that builds around the fistula, as well as the associated significant risk of facial nerve injury and post-operative facial palsy [9].

For some patients with parotid duct fistulas, especially glandular fistulas, tympanic neurectomy appears to be an effective treatment. The inhibition of parasympathetic activity caused by tympanic neurectomy has been characterized as temporary in some cases (e.g., Frey's syndrome) [10]. Low-dose radiation therapy can be used to treat parotid fistula since parotid secretions are reduced following treatment, but it is not the recommended way of treatment due to long-term side effects.

Because the gland's lobules are enclosed in the comparatively inelastic capsule, pressure dressings cause atrophy. A sustained increase in ductal pressure compresses capillaries and veins, resulting in decreased production and gland atrophy [7]. But there is no adequate proof of their efficacy in the literature.

The lack of reflex stimulation from mastication and chemical stimuli reduces parotid secretions and allows the wounded duct to heal, although this approach necessitates long-term patient compliance. Anticholinergic medications, when used alone, are associated with a variety of side effects, including urine retention, xerostomia, nausea, vomiting, visual abnormalities, and more.

Botulinum toxin A is widely used in modern medicine, however it has a long latency period [11]. Similarly, many injections are required to achieve the desired results, and the effects may not be lengthy enough to result in a complete remission of the illness. Furthermore, it is a costly procedure.

Saliva renders fibrin glue ineffective, resulting in the recurrence of the fistula [12]. Radiation therapy, the use of botulinum toxin A, the use of fibrin glue, pressure dressings, the use of antisialagogues, total parotidectomy, tympanic neurectomy, intraoral transposition of parotid duct, and other management options are available for parotid fistula which depends on the cause of fistula [7,8,10,11,[13], [14], [15], [16]]. All of the management methods described above have their own set of benefits and drawbacks. In this report, we attempted to control the parotid fistula with a warm hypertonic saline infusion for four days, and the parotid fistula spontaneously closed on the fifth day. Warm hypertonic saline injections as pressure dressings and the use of anti-sialagogues promote fibrosis of the gland in 2–3 weeks, which we believe caused the fistula to close in five days.

In sclerotherapy, several amounts of hypertonic saline have been employed. It's also been used successfully in the head and neck region for the treatment of venous abnormalities and as an alternative to surgery for varicose veins [17]. Thermodynamic and physicochemical simulations indicate that these solutions act by producing conformational denaturation of cell membrane proteins in situ, and that saline can be diluted to the point where cellular toxicity is eliminated. To boost the fibrosing property of physiologic saline, the temperature of the saline should be raised above body temperature [18].

## 4 Conclusion

In comparison to surgical intervention, conservative intervention with hot water saline for fistula closure is less expensive, causes no foreign body reaction or hypersensitive reaction in patients, is readily available, nontoxic, and nonirritant to the surrounding structures. There is also little risk of unintentional injury to the facial nerve and its branches, which would result in gland parenchyma fibrosis and spontaneous fistula closure with no sequelae.

## Trial registry number

None.

## Provenance and peer review

Not commissioned, externally peer-reviewed.

## Sources of funding

No funding was received for the study

## Ethical approval

None.

## Consent

Written informed consent was obtained from the patient for publication of this case report and accompanying images. A copy of the written consent is available for review by the Editor-in-Chief of this journal on request.

## Author contribution

RY, SS, and HBB wrote the original manuscript, reviewed, and edited the original manuscript. HBB, RY, SS, MU, AS, PBS, AD, and NP reviewed and edited the original manuscript.

## Registration of research studies

1. Name of the registry: None
2. Unique Identifying number or registration ID: None
3. Hyperlink to your specific registration (must be publicly accessible and will be checked):

## Guarantor

Sangam Shah.

## Declaration of competing interest

Authors have no conflict of interest to declare.
